# Fresh Paint

**Published:** 1884-05

**Authors:** 


### FRESH PAINT.

THE current belief among householders, that the smell of fresh lead paint is noxious, is founded on pretty general experience but is opposed by the opinion equally current among chemists, that lead compounds are not volatile. A fact recently brought to the notice of our excellent contemporary, the *Lancet*, supports the domestic theory. The basis of the useful and popular luminous paint is known to be sulphide of calcium. Now, this compound, when unprotected by varnish, glass, or some other impervious substance, is slowly acted on by the acids of the air, and sulphuretted hydrogen is evolved, which blackens lead paint. This is well known, and can easily be avoided by proper protection of the paint. But the curious thing is that unprotected luminous paint is found to be perceptibly blackened by the fumes from fresh lead paint. There seems to be only one possible explanation of this; namely, that a surface freshly covered with lead paint does actually emit some volatile compound of lead. We believe that many physicians could confirm this view from their own observations in regard to newly painted houses.

